# Inclusive Leadership and Career Sustainability: Mediating Roles of Supervisor Developmental Feedback and Thriving at Work

**DOI:** 10.3389/fpsyg.2021.671663

**Published:** 2021-07-06

**Authors:** Yang-Chun Fang, Yan-Hong Ren, Jia-Yan Chen, Tachia Chin, Qing Yuan, Chien-Liang Lin

**Affiliations:** ^1^Zhejiang University of Technology, Hangzhou, China; ^2^College of Science and Technology, Ningbo University, Ningbo, China

**Keywords:** inclusive leadership, career sustainability, supervisor developmental feedback, thriving at work, China

## Abstract

Career sustainability is a well-researched issue in academics and other sectors. Technology advancements and COVID-19 have jeopardized career sustainability. Numerous studies have explored the influence of individual characteristics on career sustainability, but few have focused on leadership. In addition, cultural factors must be considered because leadership is rooted in culture. In particular, inclusive leadership reflects traditional Chinese culture. Therefore, based on self-determination social exchange theories, we analyzed the effects of inclusive leadership on career sustainability as well as the roles of thriving at work and supervisor developmental feedback (SDF) in career sustainability. In total, 363 samples were collected from China. The results revealed that inclusive leadership improves career sustainability through SDF and thriving at work. Theoretically, our study fills the research gap and establishes a mechanism and theoretical framework for inclusive leadership and career sustainability. Practically, we offer guidance for enterprises to cultivate inclusive leadership and improve career sustainability.

## Introduction

Sustainability has become a popular research topic (Eizenberg and Jabareen, 2017; Bansal, 2019; Ilyas et al., 2020). The United Nations (UN) defines sustainability as “meeting the needs and aspirations of the present without compromising the ability to meet those needs in the future” (Brundtland et al., 1987). Scholars have begun to focus on the social dimensions of sustainability, including employee self-development, resilience, work–life balance, and job satisfaction (Manuti and Giancaspro, 2019; Abid et al., 2020; Ilyas et al., 2020), but social sustainability itself is not well-defined. The COVID-19 pandemic has caused a global unemployment crisis resulting in social unrest (Bartik et al., 2020), increasing concern about career sustainability. When people cannot obtain a stable income and quality of life through work, achieving health, safety, wellness, and well-being (the four proven determinants of social sustainability) is impossible (Staniškiene and Stankevičiute, 2018).

Career sustainability is an emerging concept. Numerous scholars have conducted theoretical research from the perspective of individuals (Herman and Lewis, 2012; Baldridge and Kulkarni, 2017; Richardson et al., 2017) but have neglected organizational factors (Barthauer et al., 2019). To benefit from sustainability, enterprises have begun to emphasize collaboration between organizations and individuals. In particular, leadership, an organizational factor, has crucial influence on career sustainability and has been widely discussed in the context of stimulating work passion (Ho and Astakhova, 2020), reducing burnout (Prastio et al., 2020), clearing identity orientation (Marstand et al., 2020), and increasing career satisfaction (Chang et al., 2020). All such factors are closely related to career sustainability, but few studies have identified a direct link between leadership and career sustainability. Therefore, we explored the influence of organizational factors on career sustainability in the context of leadership.

Traditional leadership research has focused on the individual characteristics and behaviors of leaders (Zhu and Qian, 2014). However, modern leadership research emphasizes the interaction between leaders and subordinates (Clark and Harrison, 2018; Harrison, 2018). In particular, inclusiveness is a key concept of the UN's Millennium Development Goals and the historical enlightenment of Chinese civilization (Yuan, 2007). The relationship between inclusion and sustainable development is a popular research topic in environmentalism and economics (Di Fabio and Peiró, 2018; Bijman and Wijers, 2019), and it has recently attracted increasing attention in organizational management research. Inclusive leadership advocates mutual respect between leaders and followers, common progress, and win–win cooperation; it has been proven to significantly improve employees' psychological capital, job performance, and creativity (Carmeli et al., 2010; Hirak et al., 2012; Randel et al., 2018; Zhu et al., 2019). Inclusive leadership can meet subordinates' requirements for empathy and empower them to adapt to current diversified and knowledge-oriented career trends. In addition, inclusive leadership can have a strong influence on career sustainability.

**Figure 1:**
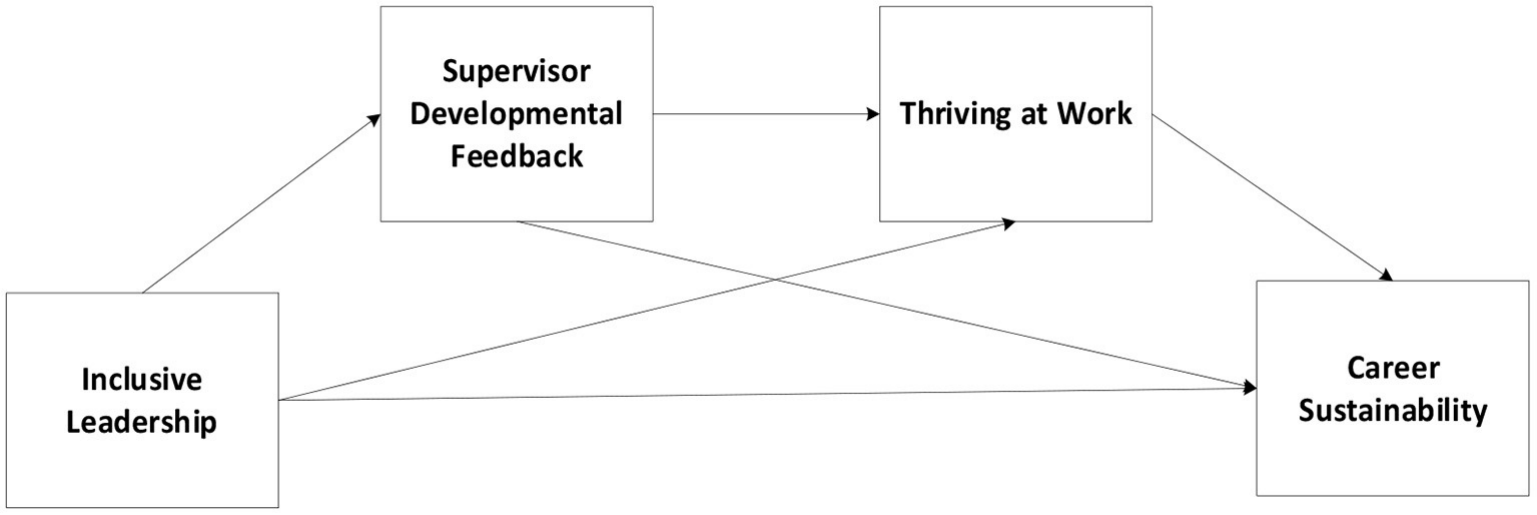
Theoretical model.

How does inclusive leadership affect career sustainability? In particular, the misalignment of the skill and job role is a critical factor leading to unemployment (Blustein et al., 2020). Technological innovation is unremitting, and individuals who endeavor to compete sustainably must continually meet increasingly higher requirements (Chin et al., 2021). According to the job characteristics model (Hackman and Oldham, 1976), feedback is a job characteristic affecting employees' work status and outcomes. Inclusive leadership emphasizes respecting, recognizing, and cultivating employees through the acknowledgment of their achievements and through the provision of developmental feedback. A new type of feedback, supervisor developmental feedback (SDF) refers to the extent to which supervisors provide their employees with helpful or valuable information that enables learning, development, and job improvement (Zhou, 2003). In particular, supervisor developmental feedback has been demonstrated to significantly increase proactive behavior, job engagement, and creativity (Eva et al., 2019; Su et al., 2019), which are conducive to continuous learning and growth. Relevant studies have suggested that a positive mental state often leads to positive subjective cognition (Abid et al., 2019). Spreitzer et al. (2012) suggested that in terms of human sustainability, “thriving” is critical for sustaining an engaged and healthy workforce. Thriving at work is crucial for ensuring an employee's positive mental state, which reflects an individual's vitality and learning experience (Spreitzer et al., 2005) and affects career development and turnover intention (Abid et al., 2015; Jiang, 2017). Therefore, we hypothesized that inclusive leadership influences career sustainability through developmental feedback and thriving at work, with self-determination theory and social exchange theory as its theoretical basis. The current study extends the research on career sustainability and inclusive leadership, further revealing the mediating role of developmental feedback and thriving at work in inclusive leadership and career sustainability. In addition, the current study provides a theoretical basis and countermeasures for incorporating inclusive leadership and ensuring career sustainability. The Theoretical model is presented as Figure 1.

## Theory and Hypotheses

### Inclusive Leadership and Career Sustainability

The concept of inclusive leadership was first proposed by Nembhard and Edmondson (2006). Hollander (2009) discussed inclusive leadership in terms of the interdependent relationship between leaders and employees, emphasizing win–win cooperation and shared vision. On the basis of Hollander's research, Carmeli et al. (2010) suggested that inclusive leadership could be judged according to openness, accessibility, and availability of communication between leaders and employees. Fang et al. (2019) defined inclusive leadership according to three aspects of the Chinese workplace: First, leaders should strive to understand their employees and tolerate their failures. Second, leaders should encourage their employees by emphasizing their training and recognizing their achievements. Third, leaders should treat employees fairly by considering their needs and sharing benefits. In the current study, we adopted this definition of inclusive leadership (Fang et al., 2019). In addition, the effects of inclusive leadership are mainly reflected in the positivity level of the individual, which has a significant and positive impact on employees' job adaptability, performance, and engagement (Hirak et al., 2012; Choi et al., 2015; Randel et al., 2018).

As societal problems related to unemployment become more prominent, people are striving to find sustainable careers (Iles, 1997). De Vos et al. (2018) conceptualized sustainable careers as “sequences of career experiences reflected through a variety of patterns of continuity over time, thereby crossing several social spaces, characterized by individual agency, herewith providing meaning to the individual.” On the basis of the findings of Newman (2011), Chin et al. (2019), and Nagy et al. (2019), defined career sustainability according to four dimensions: flexibility, renewability, integration, and resourcefulness. In the current study, we adopted this definition of career sustainability (Chin et al., 2019). Because of conceptual vagueness, the relevant literature on the antecedents and behavioral outcomes of career sustainability remains scant (Akkermans and Kubasch, 2017; Richardson et al., 2017; De Vos et al., 2018). Chin et al. (2019) suggested that factors related to the four aforementioned dimensions of career sustainability can be applied as antecedent variables of career sustainability.

Leadership plays a crucial role in an employee's career (Clark and Harrison, 2018). Studies have indicated that positive leader membership can more effectively attenuate the intent of employees to quit (Waldman et al., 2015), whereas negative leadership membership applies immense pressure on employees, causing challenges in their careers (Maruping et al., 2015). Therefore, we surmise that inclusive leadership defined by positive relationships may have a significant and positive impact on career sustainability. Specifically, inclusive leaders tolerate employees' opinions and temporary failures, consider their personal value and long-term career development, and enhance their career flexibility (Randel et al., 2018). In addition, inclusive leaders emphasize employee development, provide training opportunities for meeting changing occupational requirements, and enhance career renewability (Chin et al., 2021). Furthermore, inclusive leaders encourage employees to express opinions. When the mission of an organization is aligned with the will of individuals, career integration is enhanced (Marstand et al., 2020). Finally, inclusive leaders emphasize creating a fair organizational atmosphere and do not casually blame employees for their mistakes. This behavior helps employees feel respected, increases psychological resources (Abid et al., 2019), and reduces the risk of unemployment caused by unfair practices within the organization. Taken together, the aforementioned arguments lead to the following hypothesis:

*Hypothesis 1: Inclusive leadership is positively correlated with career sustainability*.

### Mediating Role of SDF

SDF refers to when a direct supervisor provides an employee with valuable information conducive to learning and improvement (Zhou, 2003). Few studies have investigated the antecedents of SDF, and they have only indicated that employee voice behavior can promote SDF (Sun and Zhang, 2019). Supervisors are a critical channel of feedback, and employees often adjust their behavior according to their supervisors' evaluations (Harrison, 2018). Inclusive leadership encourages supervisors to develop harmonious working relationships with their employees through active listening and providing constructive feedback, which strengthens relationships and enables employees to improve. Research on SDF outcome variables has mainly focused on the psychological cognition, behavior, and performance of individuals. Specifically, SDF promotes the intrinsic motivation to innovate (Cui and Yu, 2019) and be productive (Xu et al., 2018) by emphasizing career goals. SDF emphasizes improving skills and achieving goals, which are conducive to stimulating employees' interest in work, thereby increasing proactiveness, job satisfaction, and performance (Sommer and Kulkarni, 2012; Zheng et al., 2015; Eva et al., 2019).

According to self-determination theory, a person must continuously satisfy three basic psychological needs throughout life, namely autonomy, competence, and relatedness, to achieve optimal functioning and continuously experience personal growth (Ryan and Deci, 2000; Deci and Ryan, 2002, 2008). By providing developmental feedback, inclusive leaders can update and improve employees' competence, thereby helping employees to adapt to increasingly challenging work requirements caused by the impact of technology and to find additional opportunities for career development. SDF can also help supervisors and employees establish a connection based on mutual respect, help employees feel socially supported by others, and enhance the social and positive psychological resources for employees' career development. Thus, the following hypothesis is proposed:

*Hypothesis 2: The relationship between inclusive leadership and career sustainability is mediated by SDF*.

### Mediating Role of Thriving at Work

Spreitzer et al. (2005) defined thriving at work as “a psychological state in which an individual experiences vitality and learning at work” and was the first to introduce “thriving” in organizational settings. Recently, numerous studies have explored the antecedents of thriving at work in organizational contexts, covering not only leadership style (Mortier et al., 2016; Hildenbrand et al., 2018; Russo et al., 2018) and organizational justice (Abid et al., 2015) but also work characteristics such as innovation and timely feedback (Liu et al., 2019), autonomous decision-making (Liu and Bern-Klug, 2013), and challenging pressure (Prem et al., 2017). Inclusive leadership advocates mutual respect, common progress, and win–win cooperation between leaders and followers. This type of dependent relationship incorporates transformational, authentic, and ethical leadership styles simultaneously (Fang, 2014). Principally, employees feel acknowledged and recognized at work; therefore, they are more willing to reciprocate by working hard to improve themselves. In addition, openness and tolerance help individuals feel independent, arousing their inner vitality.

An academic consensus has been established regarding the predictive capacity of thriving at work for positive job outcomes (Abid et al., 2019). For example, thriving employees are less likely to leave their jobs (Abid et al., 2016b), miss work (Abid et al., 2016a), burn out (Spreitzer et al., 2012), and perform poorly (Elahi et al., 2019). Thriving at work promotes the sustainable development of employees through psychological and physiological benefits (Porath et al., 2012). That is, the vitality produced by thriving at work increases employee involvement in management behavior (Nelissen et al., 2017), enhances cognition and problem-solving ability through learning (Chin et al., 2020), and helps individuals determine whether their work environment is conducive to career development (Spreitzer et al., 2005), resulting in substantial adaptability in the workplace (Jiang, 2017). When employees thrive at work, they consume fewer psychological resources and higher thriving at work (Hildenbrand et al., 2018), which ensures their healthy physiological state, thereby creating a virtuous cycle that helps individuals feel energetic and motivated to confront complex challenges at work (Kark and Carmeli, 2009).

According to self-determination theory, when people are internally motivated to complete tasks, they experience positive emotions (Deci and Ryan, 2008). In addition, focusing on internal desires, such as personal growth and a sense of belonging and alliance, can more effectively fulfill people's long-term development needs than can focusing on external desires such as money, reputation, and image (Vansteenkiste et al., 2004). Inclusive leaders focus on employees' sense of organizational belonging and personal growth, respect their self-expression, and devote themselves to internalizing employees' external motivation as a sustainable positive psychological state, thereby achieving the goal of long-term cooperation and common progress between themselves and employees. Thus, the following hypothesis is proposed:

*Hypothesis 3: The relationship between inclusive leadership and career sustainability is mediated by thriving at work*.

### Serial Mediating Role of SDF and Thriving at Work

Studies have confirmed that the timeliness of feedback has a significant impact on employees' sense of thriving at work (Liu et al., 2019). According to social exchange theory, when employees receive fair and sincere treatment and care from their direct supervisors, they trust their supervisors more (Oparaocha, 2016; Tsai and Kang, 2019). Receiving more developmental feedback from supervisors helps employees more easily establish a relationship of mutual trust and increases the likelihood that employees will make changes according to their supervisors' suggestions, thus resulting in more learning behaviors. In addition, social exchange provides a wide range of inputs and emphasizes the exchange of social emotional resources (Shore et al., 2006; Song et al., 2009; Wu and Lee, 2017; Lin et al., 2019). Inclusive leadership highly emphasizes the emotional support of employees. Developmental feedback from supervisors is a positive emotional signal that expresses their willingness to communicate with employees. These leadership support behaviors are understood by employees as caring for and investment in their social and emotional needs, thus generating a sense of obligation to repay (Eisenberger et al., 2002; Nan, 2018; Roch et al., 2019), which is represented by a higher degree of vitality and work engagement. Thus, the following hypothesis is proposed:

*Hypothesis 4: The relationship between inclusive leadership and career sustainability is serially mediated by SDF and thriving at work*.

## Methods

### Sample

An online survey of employees was conducted using “wjx.cn” (https://www.wjx.cn/), a popular and professional online survey company in China (Jin et al., 2021). “wjx.cn” has developed a database of over 2.6 million employees covering different companies and industries in china, employees voluntarily filled out the questionnaire through the link on the website. After completing the questionnaire, they received a monetary reward of a random amount (¥ 1-5). Previous research on Chinese employees' organizational behavior has also used “wjx.cn” as a data collection tool (Ren et al., 2020). One data sample consisted of a single questionnaire completed by one person. We received 385 questionnaires between August and November 2020, and 22 were excluded because they did not meet completeness and normative requirements (Su et al., 2021). In total, 363 valid questionnaires were obtained, a valid response rate of 94.28%. Table 1 presents the demographic data of the participants.

**Table 1:** Demographic data.

**Characteristic**	**Categories**	**Frequency**	**Percentage**
Gender	Male	158	43.5%
	Female	205	56.5%
Age	Under 30 years	148	40.8%
	30–39 years	170	46.8%
	40–49 years	39	10.7%
	50–59 years	4	1.1%
	60 years and over	2	0.6%
Education	High school and below	12	3.3%
	College	49	13.5%
	Bachelor	252	69.4%
	Master	47	12.9%
	Doctor	3	0.8%
Length of employment	Under 5 years	169	46.6%
	5–9 years	136	37.5%
	10–14 years	40	11%
	15–19 years	9	2.5%
	20 years and over	9	2.5%
Nature of enterprise	State-owned enterprise	67	18.5%
	Private enterprise	237	65.3%
	Foreign capital enterprise	24	6.6%
	Government-affiliated institutions	14	3.9%
	Others	21	5.8%

### Measures

The Inclusive Leadership (IL) scale developed by Fang et al. (2019) contains 11 items, including “In my work, the leaders actively ask my opinions and thoughts,” “The leaders treat us equally and always adhere to certain commonly recognized principles,” and “When employees make mistakes, the leaders express emotional understanding and suggestions for improvement.” We used this scale because it is consistent with the design of our study.

The Supervisor Developmental Feedback (SDF) scale developed by Zhou (2003) was also used because it is consistent with the design of our study. The scale contains three items: “While giving me feedback, my supervisor focuses on helping me to learn and improve,” “My immediate supervisor never gives me developmental feedback,” and “My supervisor provides me with useful information on how to improve my job performance.”

In addition, the thriving at work (TW) scale designed by Porath et al. (2012) is a development feedback scale commonly used in relevant research and has been proven to have high reliability and validity. It measures thriving according to two criteria: “I feel alive and vital” and “I feel alert and awake.”

The career sustainability (CS) scale developed by Chin et al. (2020) is relatively new but is consistent with the design of our study. It measures career sustainability according to the following criteria: “My career makes me feel a bright future,” “My career helps me develop my potential to complete the task,” and “My career cultivates my ability to master different information.”

Research suggests that career sustainability is affected by personal factors such as gender, education, length of employment, and nature of work (Chin et al., 2020). Thus, we applied these factors as control variables in our analysis of career sustainability. In the questionnaire, for gender, the male gender was encoded as 1 and the female gender as 0; for education level, high school or below to doctorate were scaled from 1 to 5; and for the length of employment and nature of work, five categories scaled from 1 to 5 were included for analysis.

### Procedures

The respondents in the research indicated their intention to participate in this study and completed the questionnaire voluntarily. All the respondents were anonymous and agreed to participate in the survey of this study in order to collect data. The survey was conducted in Chinese. When conducting the online survey, we explained the confidentiality of the survey process. None of the questions involved confidential information, and individual respondents completed the survey anonymously. Therefore, all the respondents were voluntary and their personal information and opinions were confidential and did not relate to any sensitive issues.

### Common Method Variance

Common method variance is related to the measurement method and does not originate from the construct represented by the measurement item itself (Williams and Anderson, 1994; Williams and Brown, 1994; Podsakoff et al., 2003), possibly resulting in measurement errors. To reduce common method variance, the current study adopted two methods. First, for questionnaires, the scale was paginated, and an appropriate rest time was provided between answering each page. Thus, the resulting time difference reduced the influence of common method variance caused by the same continuity scale (Podsakoff et al., 2003). Second, Harman's single-factor test was used to verify whether common method variance occurred (Podsakoff et al., 2003). Exploratory factor analysis indicated that the first factor explained only 41.794% of the variance, which was lower than the 50% threshold (Shiau and Luo, 2012). Therefore, no significant common method variance was observed; it was within the acceptable range.

## Results

### Measurement Model

SmartPLS 3.3.3 package was used to test the hypotheses in our research model (Ringle et al., 2015). A measurement model was employed to determine reliability, convergent validity, and discriminant validity (Liang and Shiau, 2018; Shiau et al., 2020). SEM–PLS was employed for the measurement and structural model analysis mainly because it, compared with covariance-based SEM, involves analyzing the complex relationships between observed and latent variables. SEM–PLS has been widely adopted for analysis in research on marketing management, information management, organizational management, human resources management, and tourism management (Yang and Lin, 2014; Suen et al., 2019; Jin et al., 2021; Su et al., 2021).

For reliability, internal consistency was ensured by determining the composite reliability of the constructs (Fornell and Larcker, 1981). Cronbach's α values for each dimension ranged from 0.812 to 0.942 (SDF and IL, respectively), which were higher than the recommended value of 0.7. Combined reliability ranged from 0.889 to 0.950 (SDF and IL, respectively), which were all higher than 0.8. In addition, the consistent partial least squares (PLS) method was used to correct the estimate of the measured structure with a new reliability coefficient, rho_A, which ranged from 0.816 to 0.945 (SDF and IL, respectively). These results confirm the high internal consistency of the measurements (Dijkstra and Henseler, 2015; Hair et al., 2017, 2019).

In terms of convergent validity, the factor loadings of all items were significant (>0.7). The average variance extracted (AVE) values were higher than 0.5, ranging from 0.595 to 0.727 (IL and TW, respectively). Therefore, the convergent validity of these measures was satisfactory. The discriminant validity of the constructs was evaluated using the approach described by Fornell and Larcker (1981) and the heterotrait–monotrait (HTMT) method (Henseler et al., 2014). In the way of Fornell and Larcker (1981), all square roots of the AVE values were higher than all correlation coefficients, indicating the satisfactory discriminant validity of the measures (Fornell and Larcker, 1981; Chin and Marcoulides, 1998). Another way is, Henseler et al. (2014) proposed the heterotrait–monotrait (HTMT) ratio of the correlations and suggested 0.90 as a threshold value for structural models with constructs. In the current study, the values ranged from 0.423 to 0.614, which indicated that discriminate validity was established for all constructs of the model. The data presented in Tables 2, 3 satisfy the two aforementioned criteria, indicating high discriminant validity.

**Table 2:** Discriminant validity analysis (Fornell and Larcker).

**Construct**	**IL**	**SDF**	**TW**	**CS**
IL	0.797			
SDF	0.463	0.853		
TW	0.569	0.510	0.782	
CS	0.582	0.374	0.545	0.772

*IL, Inclusive Leadership; SDF, Supervisor Developmental Feedback; TW, Thriving at Work; CS, Career Sustainability*.

**Table 3:** Discriminant validity analysis (HTMT).

**Construct**	**IL**	**SDF**	**TW**	**CS**
**IL**				
SDF	0.525			
TW	0.604	0.584		
CS	0.614	0.423	0.579	

*IL, Inclusive Leadership; SDF, Supervisor Developmental Feedback; TW, Thriving at Work; CS, Career Sustainability*.

Henseler et al. (2014) introduced standardized root mean squared residual (SRMR) as the square root of the sum of squared differences between the model-implied and empirical correlation matrix. A value lower than 0.10 is considered to denote a good fit (Henseler et al., 2014). The SRMR in the current study was 0.045, indicating a favorable model. Regarding multicollinearity, according Hair et al. (2017), value tolerance has a variance inflation factor (VIF) value below 5. Table 4 presents all construct VIF values ranging from 1.469 to 1.699, which indicate that the results meet the requirements.

**Table 4:** Confirmatory factor analysis results for the measured variables.

**Construct**	**Items**	**Factor loading**	**α**	**rho_A**	**CR**	**AVE**	**VIF**
Inclusive Leadership (IL)	IL1	0.792	0.942	0.945	0.950	0.635	1.595
	IL2	0.812					
	IL3	0.807					
	IL4	0.792					
	IL5	0.804					
	IL6	0.754					
	IL7	0.748					
	IL8	0.768					
	IL9	0.814					
	IL10	0.845					
	IL11	0.824					
Supervisor Developmental Feedback (SDF)	SDF1	0.822	0.812	0.816	0.889	0.727	1.469
	SDF2	0.862					
	SDF3	0.874					
Thriving at Work (TW)	TW1	0.794	0.929	0.929	0.940	0.611	1.699
	TW2	0.79					
	TW3	0.766					
	TW4	0.780					
	TW5	0.773					
	TW6	0.769					
	TW7	0.809					
	TW8	0.755					
	TW9	0.769					
	TW10	0.811					
Career Sustainability (CS)	CS1	0.719	0.938	0.939	0.946	0.595	DV
	CS2	0.805					
	CS3	0.783					
	CS4	0.769					
	CS5	0.769					
	CS6	0.767					
	CS7	0.761					
	CS8	0.77					
	CS9	0.787					
	CS10	0.793					
	CS11	0.775					
	CS12	0.757					

*IL, Inclusive Leadership; SDF, Supervisor Developmental Feedback; TW, Thriving at Work; CS, Career Sustainability*.

### Structural Model

**Figure 2:**
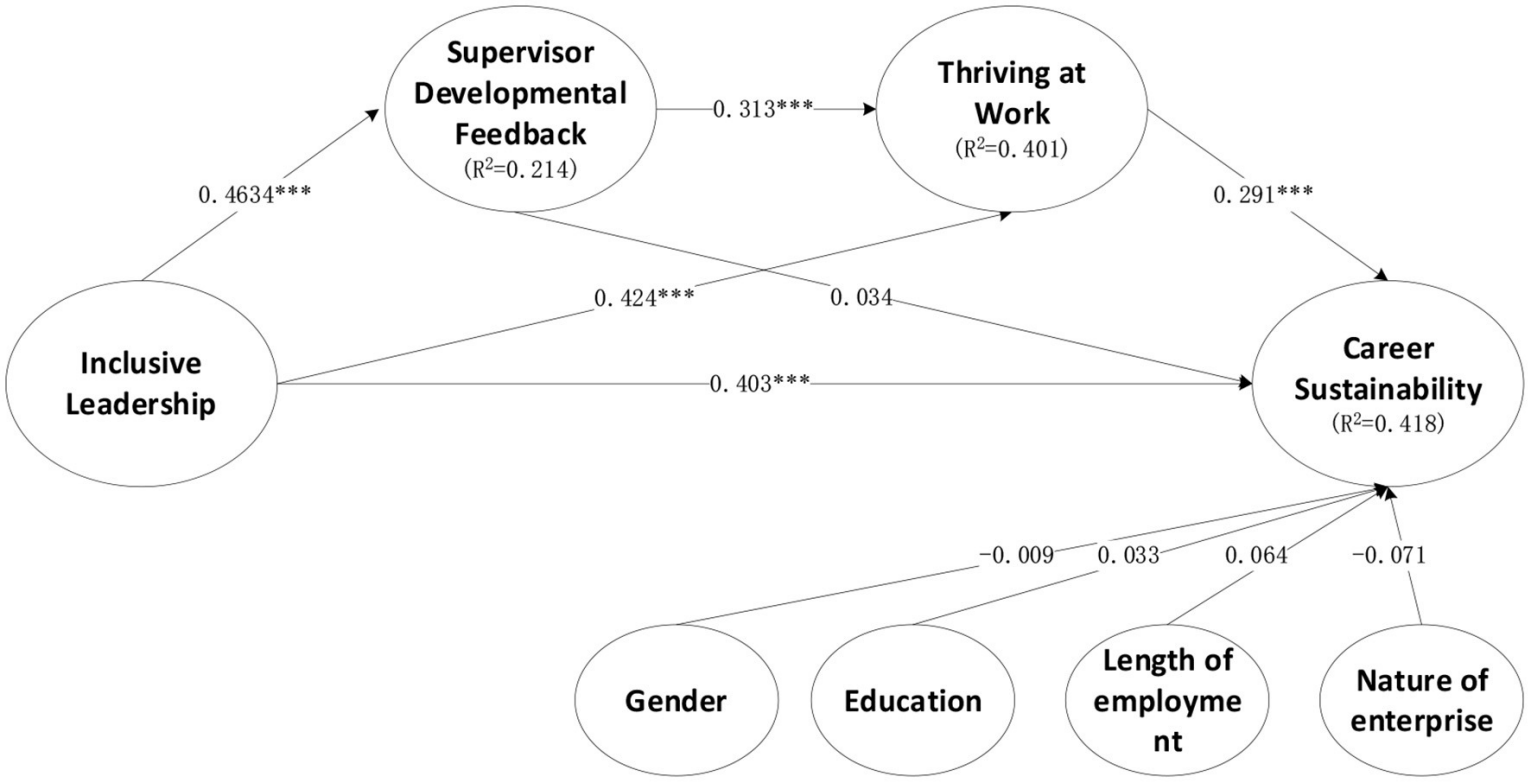
PLS results of the research model.

To test the hypotheses, the bootstrap resampling method in SmartPLS was used to evaluate the PLS results, and the responses were resampled 5,000 times (Hair et al., 2017). Table 5 presents the results. The overall *R*^2^ value was 0.418, and the results support hypotheses H1, H3, and H4. The results suggest that inclusive leadership had a positive effect on career sustainability (H1). In addition, the mediating role of thriving at work in the relationship between inclusive leadership and career sustainability was verified (H2), but the mediating role of SDF was not verified. Furthermore, the relationship between inclusive leadership style and career sustainability was serial-mediated by SDF and thriving at work (H4). Figure 2 presents PLS results of the research model.

**Table 5:** Results of the serial mediation test.

**Indirect effect**	**Effect**	**SE**	***T*-value**	***P*-value**	**Result**
Total indirect effect	0.181	0.065	2.774	0.006	Significant
Path 1: IL → SDF → CS	0.016	0.027	2.567	0.557	Not significant
Path 2: IL → TW → CS	0.123	0.048	3.763	0.011	Significant
Path 3: IL → SDF → TW → CS	0.042	0.013	3.179	0.002	Significant

## Discussion

### Implications for Theories

Most relevant studies on the antecedents of career sustainability are qualitative studies conducted from the perspective of individuals (Herman and Lewis, 2012; Baldridge and Kulkarni, 2017). Only one study explored the antecedents of career sustainability in terms of the organizational context (Tordera et al., 2020). We consider the organizational context and individual characteristics to be essential to career sustainability, and our findings support this. To a certain extent, we explored the interaction influence between inclusive leadership, SDF (organizational situations), and thriving at work (individual psychological characteristics) on career sustainability. The current study elucidated the antecedent variables of career sustainability at individual and organizational levels and confirmed the theoretical inferences of Chin et al. (2019) on the antecedent variables of career sustainability in terms of flexibility, renewability, integration, and resourcefulness.

In addition, the current study expands applications for the theory of inclusiveness and sustainability in terms of organizational behavior. The relevant literature has revealed the crucial role of inclusiveness in sustainable economic growth, ecological sustainability, and sustainable urban development, but few studies have been examined organizational behavior. The current study innovatively introduces the concepts of inclusiveness and sustainability into organizational behavior, further confirming the inseparable relationship between inclusiveness and sustainability. The application of inclusiveness in organizational behavior is mainly reflected in leadership. The impact of inclusive leadership on employee creativity, performance, and turnover rate (Fang, 2014; Randel et al., 2018; Zheng et al., 2018; Javed et al., 2019; Kim and Moon, 2019; Ye et al., 2019) has been widely acknowledged, but research on sustainability in organizational behavior is mainly reflected in sustainable human resource management (Ren et al., 2017). Studies have identified a positive relationship between leadership and green human resource management (GHRM) (Jia et al., 2018), and the relationship between inclusive leadership and GHRM should be explored in future studies.

Furthermore, in terms of the cross-disciplinary aspects of management and psychology, we revealed a mediating role of SDF and thriving at work. Specifically, we found that the relationship between inclusive leadership and career sustainability was not separately mediated by SDF but rather serially mediated through thriving at work. This may be because in the long term, career sustainability is easily affected by external environments, and employees generally have a subjective plan for their careers; therefore, developmental feedback does not considerably influence career sustainability through cognition ways. However, SDF can construct an image of organizational support for employees to reference in numerous challenging future work scenarios (Blustein, 2011). Currently, few studies have been conducted on SDF, and they have mainly focused on outcome variables. To a certain extent, the current study extends the research on the antecedents and outcome variables of SDF. Studies on thriving first appeared in the psychology research field. Recently, an increasing number of studies on thriving have been conducted in the management research field. However, some scholars believe that the roles of leadership (Paterson et al., 2013) and personal characteristics in the promotion of thriving (Nawaz et al., 2018) are insufficiently studied. This paper identifies the positive impact of inclusive leadership on thriving at work and further confirms the conclusions of Zeng et al. (2020).

### Implications for Practice

First, enterprises should cultivate inclusive leadership. The impact of inclusion on sustainability has been widely recognized, and enterprises should recognize the positive role of the inclusive leadership style in developing career sustainability. Specifically, leaders should be receptive to employees and tolerate their mistakes with encouragement and support. In addition, leaders should respect and recognize their employees, focus on employee development, and praise employees for their achievements, rather than envy them. Moreover, leaders should consider the needs of employees, treat them fairly and justly, and share benefits with them.

Second, developmental feedback should be effectively utilized. Feedback has been widely used in enterprises as an effective management tool. Constructing effective feedback has always been the focus of academic and practical discussions (Li et al., 2011). As mentioned, we found that the role of SDF in career sustainability is mainly reflected on the psychological level. Therefore, we suggest that when providing developmental feedback to employees, performance and instrumental guidance should be minimized; rather, psychological support should be emphasized, and employees' internal motivation should be promoted by satisfying their needs for autonomy, a sense of belonging, and competency.

Third, enterprises should actively emphasize the psychological state of their employees. Human sustainability is the basis of career sustainability, and healthy psychological and physiological conditions are the basis of human sustainability. Numerous studies have identified a close relationship between the mental state and physical health (Spreitzer et al., 2012). However, most enterprises have focused on performance and have neglected the mental state of their employees. Because of the crucial role of the positive mental state in career sustainability, managers must fully understand the psychological needs of their employees and must effectively respond to changes in their mental states by promoting thriving at work. Maintaining employees' energy and enthusiasm for learning can be achieved by creating an atmosphere of trust and respect as well as by providing employees with healthy interactions and positive emotional resources.

### Limitations and Future Directions

First, cross-sectional data were adopted in the current study, and each questionnaire was completed by employees themselves; therefore, accurately and rigorously measuring the causal relationship between variables is difficult. Future studies should consider further reducing measurement errors by issuing questionnaires at different time points and from different sources or by enhancing the control variables during experimental research.

Second, the sample population mainly consisted of employees from Chinese enterprises. In the future, the sample population can include individuals from various countries and industries. A cross-layer research method can also be adopted to improve the accuracy and external validity of the data.

Third, the current study only explored one of the ways that inclusive leadership can have a positive influence on career sustainability. Other influences paths may have not been discovered.

Finally, the differences in individual personality characteristics of employees may also affect the significance of the study. In the future, moderating variables can be added to explore the establishment boundary of the serial mediating effect.

## Conclusions

Research has revealed that inclusive leadership plays a crucial role in promoting the career sustainability of employees. We further investigated the serial mediating effect of SDF and thriving at work in terms of cross-level interactions between individual characteristics and organizational contexts. Therefore, the serial mediating effect helped us identify the key factors of career sustainability and improved our understanding of how enterprises can fully apply these factors to improve career sustainability. More importantly, the results may help researchers outside China conduct similar studies and explore the adaptability of the research in different cultural contexts. In general, sustainability has become the focus of global practice and academia. Individual sustainable development is the original source of sustainable development in society and among enterprises; therefore, leaders must understand how to achieve corporate sustainability by helping employees improve their career sustainability. Based on valuable empirical evidence, a feasible approach was identified, which can be applied by enterprises. Furthermore, this paper indicates how enterprises can more effectively fulfill their social responsibilities and echoes the common pursuit of humans for the sustainable development of society.

## Data Availability Statement

The original contributions presented in the study are included in the article/supplementary material, further inquiries can be directed to the corresponding author/s.

## Author Contributions

Y-CF conceived and designed the research and provided guidance throughout the entire research process. Y-HR wrote the main part of the manuscript. J-YC collected the data and written the methods section. TC and C-LL helped translating and offered modification suggestions. QY participated in the literatures collecting and organizing. All authors listed have made a substantial, direct and intellectual contribution to the work, and approved it for publication.

## Conflict of Interest

The authors declare that the research was conducted in the absence of any commercial or financial relationships that could be construed as a potential conflict of interest.
